# Degradable hydrogel fibers encapsulate and deliver metformin and periodontal ligament stem cells for dental and periodontal regeneration

**DOI:** 10.1590/1678-7757-2022-0447

**Published:** 2023-04-28

**Authors:** Jingyao YIN, Qian LEI, Xinghong LUO, Tao JIANG, Xianghui ZOU, Abraham SCHNEIDER, Hockin H. K. XU, Liang ZHAO, Dandan MA

**Affiliations:** 1 Southern Medical University Stomatological Hospital Department of Endodontics Guangzhou Guangdong China Southern Medical University, Stomatological Hospital, Department of Endodontics, Guangzhou, Guangdong, China.; 2 Southern Medical University School of Stomatology Guangzhou Guangdong China Southern Medical University, School of Stomatology, Guangzhou, Guangdong, China.; 3 University of Maryland School of Dentistry Department of Oncology and Diagnostic Sciences Baltimore Maryland USA University of Maryland School of Dentistry, Department of Oncology and Diagnostic Sciences, Baltimore, Maryland, USA.; 4 University of Maryland Dental School Department of Advanced Oral Sciences and Therapeutics Baltimore Maryland USA University of Maryland Dental School, Department of Advanced Oral Sciences and Therapeutics, Biomaterials and Tissue Engineering Division, Baltimore, Maryland, USA.; 5 University of Maryland School of Medicine Marlene and Stewart Greenebaum Cancer Center Baltimore Maryland USA University of Maryland School of Medicine, Marlene and Stewart Greenebaum Cancer Center, Baltimore, Maryland, USA.; 6 University of Maryland School of Medicine Center for Stem Cell Biology and Regenerative Medicine Baltimore Maryland USA University of Maryland School of Medicine, Center for Stem Cell Biology and Regenerative Medicine, Baltimore, Maryland, USA.; 7 Shunde Hospital Department of Trauma and Joint Surgery Guangzhou Guangdong China Shunde Hospital, Department of Trauma and Joint Surgery, Guangzhou, Guangdong, China.; 8 Southern Medical University Nanfang Hospital Department of Orthopaedic Surgery Guangzhou Guangdong China Southern Medical University, Nanfang Hospital, Department of Orthopaedic Surgery, Guangzhou, Guangdong, China.

**Keywords:** Periodontal, Stem cell, Metformin, Osteogenic, Tissue engineering

## Abstract

Human periodontal ligament stem cells (hPDLSCs) are promising cells for dental and periodontal regeneration.

**Objective:**

This study aimed to develop novel alginate-fibrin fibers that encapsulates hPDLSCs and metformin, to investigate the effect of metformin on the osteogenic differentiation of hPDLSCs, and to determine the regulatory role of the Shh/Gli1 signaling pathway in the metformin-induced osteogenic differentiation of hPDLSCs for the first time.

**Methodology:**

CCK8 assay was used to evaluate hPDLSCs. Alkaline phosphatase (ALP) staining, alizarin red S staining, and the expression of osteogenic genes were evaluated. Metformin and hPDLSCs were encapsulated in alginate-fibrinogen solutions, which were injected to form alginate-fibrin fibers. The activation of Shh/Gli1 signaling pathway was examined using qRT-PCR and western blot. A mechanistic study was conducted by inhibiting the Shh/Gli1 pathway using GANT61.

**Results:**

The administration of 50 μM metformin resulted in a significant upregulation of osteogenic gene expression in hPDLSCs by 1.4-fold compared to the osteogenic induction group (P < 0.01), including ALP and runt-related transcription factor-2 (RUNX2). Furthermore, metformin increased ALP activity by 1.7-fold and bone mineral nodule formation by 2.6-fold (P<0.001). We observed that hPDLSCs proliferated with the degradation of alginate-fibrin fibers, and metformin induced their differentiation into the osteogenic lineage. Metformin also promoted the osteogenic differentiation of hPDLSCs by upregulating the Shh/Gli1 signaling pathway by 3- to 6- fold compared to the osteogenic induction group (P<0.001). The osteogenic differentiation ability of hPDLSCs were decreased 1.3- to 1.6-fold when the Shh/Gli1 pathway was inhibited, according to ALP staining and alizarin red S staining (P<0.01).

**Conclusions:**

Metformin enhanced the osteogenic differentiation of hPDLSCs via the Shh/Gli1 signaling pathway. Degradable alginate-fibrin hydrogel fibers encapsulating hPDLSCs and metformin have significant potential for use in dental and periodontal tissue engineering applications.

**Clinical Significance:**

Alginate-fibrin fibers encapsulating hPDLSCs and metformin have a great potential for use in the treatment of maxillofacial bone defects caused by trauma, tumors, and tooth extraction. Additionally, they may facilitate the regeneration of periodontal tissue in patients with periodontitis.

## Introduction

Periodontitis is an inflammatory illness of the periodontium that includes the gingiva, alveolar bone, periodontal ligament (PDL), and cementum. It is characterized by inflammation and alveolar bone loss, and it may lead to tooth loss if left untreated. Ideally, regenerative periodontology aims to regenerate these lost tissues to their original architecture and function, which is a challenging task. Several approaches for periodontal regeneration have been explored, including the use of gingival margin-derived stem/progenitor cells combined with IL-1ra short term releasing HA hydrogel synthetic extracellular matrix, which has shown periodontal regenerative potential,^1^ and PDLSCs encapsulated in TGF-β3-loaded RGD-modified alginate microspheres, which are promising candidates for regeneration.^2^ With the rapid development of cell biology and materials science, the technology of encapsulating drugs and cells in materials has become a research hotspot in the healing and regeneration of alveolar bone defects. With the rapid development of cell biology and materials science, the technology of encapsulating drugs and cells in materials has become a research focus in the field of alveolar bone defects healing and regeneration.^1,2^

In recent years, an increasing number of studies have confirmed that metformin is an antihyperglycemic biguanide compound, and has many biochemical activities, such as anti-aging, anti-tumor, anti-inflammatory, and anti-cardiovascular diseases.^3-5^ Furthermore, metformin stimulates the osteogenic/dentinogenic differentiation of various mesenchymal stem cells (MSCs), such as adipose stromal cells,^6^ dental pulp stem cells (DPSCs),^7^ human periodontal ligament stem cells (hPDLSCs),^8^ and induced pluripotent stem cell-derived MSCs.^9^ However, few studies have reported the combined application of metformin and hPDLSCs for the regeneration of alveolar bone defects.

Biomaterials loaded with metformin have been shown to promote cellular osteogenesis and dentinogenesis, such as nanosphere-laden photocrosslinkable gelatin hydrogels, tricalcium silicate-based cements, polydopamine-templated hydroxyapatite, and resin.^10-13^ Some of these materials, however, induced the production of reactive oxygen species. As a result, cells are damaged and apoptosis occurs. These systems are unable to carry cells and drugs at the same time, which is not conducive to drug and stem cell delivery to bone defects.

As a highly hydrated natural material with good biocompatibility, alginate hydrogels are expected to be able to carry drugs and cells at the same time. Therefore, we developed degradable alginate-fibrin hydrogel fibers to encapsulate hPDLSCs and metformin simultaneously. We hypothesized that the degradation process of the hydrogel fibers would lead to the sustained release of metformin, which, in combination with the progressive proliferation and osteogenic differentiation of hPDLSCs, could effectively promote the regeneration of alveolar bone.

Hedgehog is a secreted signaling molecule that regulates all stages of embryonic development and the production of many tissues and organs, including tooth development.^14^ The high expression of Gli protein indicates the activation of the Shh signaling pathway. Shh has been shown to stimulate adult PDL-derived Stro-1^+^ cells to produce Gli1 and PTC-1, and can selectively promote cell proliferation.^15^ Gli1^+^ cells are pluripotent stem cells in the periodontal tissue of adult mice that can form alveolar bone, cementum, and PDL.^16^ Previous studies have demonstrated that Shh/Gli1 signaling pathway is involved in osteogenic differentiation of DPSCs.^17^ However, as far as we know, there has been no previous report on the role of Shh signaling pathway in the osteogenic differentiation of hPDLSCs induced by metformin.

Therefore, this study investigated the effect of Shh/Gli1 pathway on metformin-induced osteogenesis in hPDLSCs delivered via degradable hydrogel fibers for the first time. This study sought to develop novel degradable alginate-fibrin hydrogel fibers that encapsulates hPDLSCs and metformin for dental and periodontal tissue regeneration, to investigate the effects of metformin on the proliferation and osteogenic differentiation of hPDLSCs, and to determine the regulatory role of the Shh/Gli1 signaling pathway in metformin-induced osteogenic differentiation of hPDLSCs for the first time.

## Methodology

### hPDLSC culture and identification

PDL tissues were collected from human adults (aged 18-25) who had their healthy wisdom teeth or premolars extracted due to orthodontic therapy, which was approved by NanFang Hospital, Southern Medical University. All patients or their respective guardians provided an informed consent form. hPDLSCs were prepared using the methods described in the previous studies with minor modifications.^18^ PDL tissues were isolated from the middle third of the root surface, cut into tiny pieces, and digested with 3 mg/mL collagenase I (Solaibao, Beijing, China) for 20 minutes in a 5% CO_2_ incubator at 37^0^. The digested tissues were then placed on culture dishes with Dulbecco’s modified Eagle’s medium (DMEM) (GIBCO, Grand Island, NY, USA) supplemented with 10% fetal bovine serum (ExCell Bio, Shanghai, China) and 1% penicillin/streptomycin (GIBCO, Grand Island, NY, USA) at 37^0^ with 5% CO_2_. Individual hPDLSCs were observed after 5-7 days. Multiple colonies were collected to enrich hPDLSCs, and cells at passages 3-6 were employed in the studies. The expression of CD29, CD90, CD34 and CD45 on the surface of hPDLSCs was determined using flow cytometry (FACSCalibur, BD, USA).

### hPDLSC viability and proliferation assays

hPDLSCs were plated in 96-well culture plates at a density of 3x10^3^ cells/well and incubated with 0 μM, 30 μM, 50 μM, or 100 μM metformin for CCK8 assay. Cells were detected on Days 1, 3, 5, and 7. After culture, 90 μL of DMEM and 10 μL of CCK8 reagent (DOJINDO, Kumamoto, Japan) were added to each well of the culture plate, and the cells were cultured at 37 °C for 1 hour in the dark. The absorbance was measured at 450 nm using a SpectraMax M5 multifunctional microplate reader (BD Falcon, San Jose, USA).^19^ All the experiments were performed in triplicate.

### Alkaline phosphatase (ALP) activity staining

hPDLSCs were seeded in 24-well plates in 500 μL of complete culture medium, grown to 70% confluence, and then cultured for 14 days in osteogenic induction medium. ALP activity was detected using the ALP Assay kit (QuantiChrom, BioAssay Systems, Hayward, CA, USA) with p-Nitrophenylphosphate (pNPP) as a substrate and BCIP/NBT Alkaline Phosphatase Kit (Biyuntian, Shanghai, China), following the manufacturer’s instructions.^20^ All experiments were performed in triplicate.

### Bone mineralization assays

hPDLSCs were seeded in 24-well plates in 500 μL of complete culture medium, grown to 70% confluence, and then cultured for 14 days in osteoinduction medium. Each well was fixed for 30 minutes with 4% paraformaldehyde, washed three times with phosphate-buffered saline (PBS), and stained with alizarin red S (Millipore, Burlington, USA). To test the generated minerals, Xylenol orange (XO) staining (Sigma, Saint Louis, USA) was performed by measuring red fluorescence. Cells were treated with 2 mL of osteogenic induction medium containing 100 μL of XO overnight after 14 days of osteogenic culture.^21^ An epifluorescence microscope (Eclipse TE-2000S, Nikon, Tokyo, Japan) was used to examine bone mineral nodule formation. All experiments were performed in triplicate.

### Quantitative real-time polymerase chain reaction (qRT-PCR)

**Figure 1:**
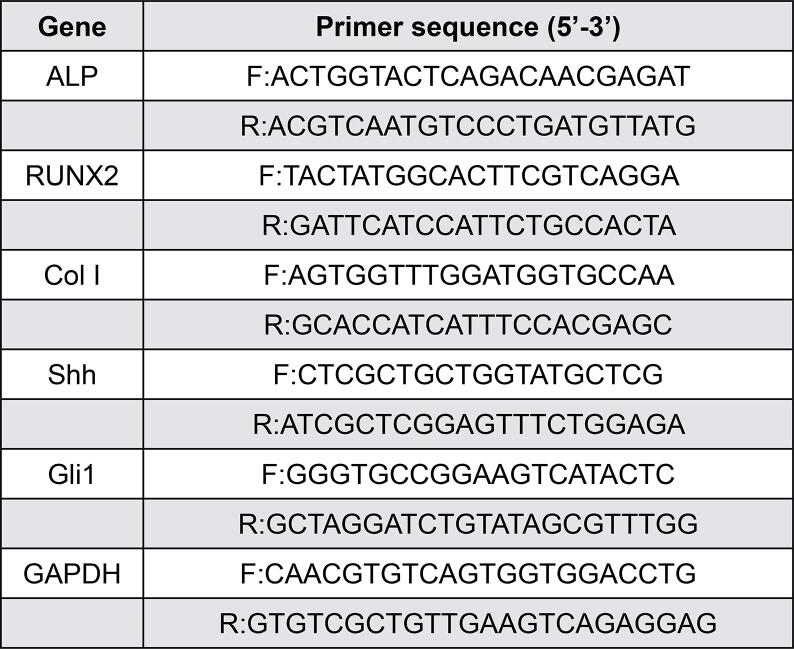
Primer sequences for qRT-PCR

Total RNA was isolated from cells using TRIzol Reagent (Takara, Shiga, Japan). An aliquot of 1000 ng of RNA was reverse transcribed to cDNAs using Takara PrimeScript Reverse Transcriptase (Takara, Shiga, Japan). A QuantStudio 5 Real-time PCR machine (Thermo Fisher, Waltham, MA, USA) was used to perform qRT‒PCR with SYBR Premix DimerEraser^TM^ (Takara, Shiga, Japan). Figure 1 lists the primer sequences utilized in the tests. Three separate experiments were performed using human glyceraldehyde-3-phosphate dehydrogenase (GAPDH) as a housekeeping gene to normalize the mRNA levels. The 2^-^ approach was used to calculate the relative expression of target genes. All the experiments were performed in triplicate.

### Western blot analysis

Cell lysates were produced and western blot analysis was performed as previously described.^17^ The membranes were subsequently treated with the following primary antibodies: ALP (Abcam, ab108337), Col I (Abcam, ab34710), runt-related transcription factor-2 (RUNX2) (Abcam, ab23981), GAPDH (Bioworld, BS72410), Gli1(Abcam, ab134906), and Shh (Abcam, ab53281). All the experiments were performed in triplicate.

### Encapsulation of cells and metformin in alginate-fibrin fibers

Alginate (64% guluronic acid, MW = 75,000-220,000 g/mol, ProNova, Oslo, Norway) was oxidized to 7.5% using reported procedures to increase the degradability of the hydrogel.^22^ The 7.5% oxidized alginate was mixed with a 155 mM sodium chloride solution to prepare a 2% sodium alginate solution. Then, fibrinogen from bovine plasma (Sigma, Saint Louis, USA) was added at a concentration of 0.4%.^23^ hPDLSCs with and without metformin were added to the alginate-fibrinogen solution at a density of 1×10^6^ cells/mL with a metformin concentration of 50 μM. Cells with or without the metformin solution were extruded into a 100 mL solution containing 100 mmol/L calcium chloride (Sigma) and 1 NIH units per mL of thrombin (Sigma) at a rate of 6 mL/min with a 27-gauge needle (with a 210 μm inner diameter) attached to a syringe pump (NE-300, New Era Pump Systems, Farmingdale, NY). The reaction between fibrinogen and thrombin occurred when the alginate-fibrinogen solution was streamed into the bath waters, resulting in fibrin fiber formation. The alginate-fibrin fibers were incubated in the bath for 20 minutes for cross-linking.^24^ The alginate-fibrin fibers were then rinsed twice with PBS. An optical microscope (Eclipse TE-2000S, Nikon, Melville, NY) was used to examine the fibers.

The alginate-fibrin fibers incubated with 0.4% fibrinogen lost their integrity and released most of the encapsulated cells on day four, according to our previous study.^23^ Therefore, three groups were tested by performing live/dead staining and ALP activity assays, and alizarin red S staining of the second and third groups was performed:

1. alginate-fibrin fiber-encapsulated hPDLSCs cultured in growth medium;
2. alginate-fibrin fiber-encapsulated hPDLSCs cultured in osteogenic medium; and
3. alginate-fibrin fiber-encapsulated hPDLSCs with 50 μM metformin cultured in osteogenic medium.

### Statistical analysis

The area fractions of ALP staining, alizarin red S staining, and xylenol orange staining were calculated using Image-Pro Plus software. The western blot results were calculated using ImageJ software. The data were statistically evaluated utilizing GraphPad Prism software (GraphPad, USA). An unpaired t test was used to assess significant differences. All data are presented as the means ± SEM, and the significance level was set to *P*<0.05 based on results from at least three independent samples.

## Results

### Metformin was not toxic to hPDLSCs

**Figure 2:**
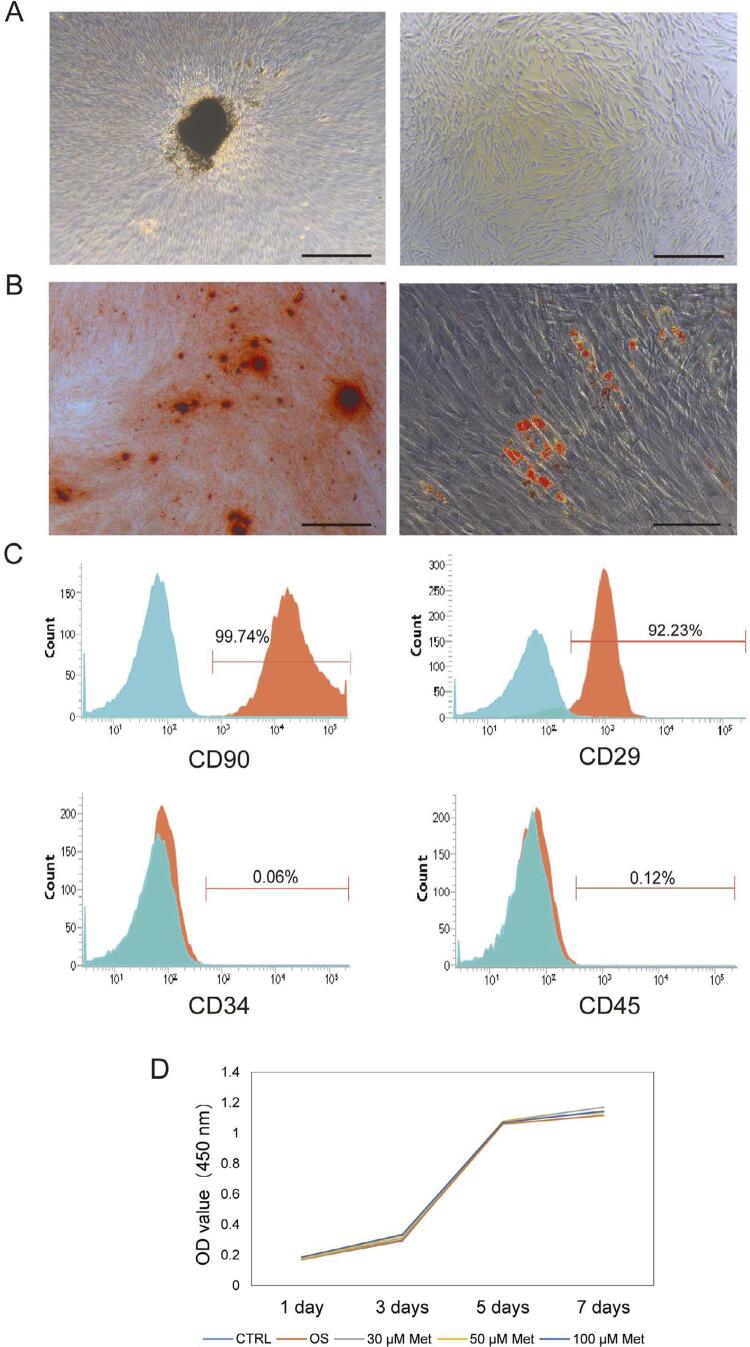
Identification of hPDLSCs and the effect of metformin treatment on hPDLSCs viability. A, Primary culture and subculture of hPDLSCs. (scale bar=500 μm) B, hPDLSCs osteogenic and adipogenic differentiation (scale bar=500 μm) C, hPDLSCs were identified using flow cytometry (Mesenchymal stem cell markers: CD29 and CD90; Hematopoietic stem cell markers: CD34 and CD45) D, Cell viability by a CCK8 assay after treating with different concentrations of metformin (P>0.05). (CTRL: control; OS: osteogenic induction; Met: metformin + osteogenic induction) (n=3)

hPDLSCs were successfully isolated from extracted human teeth (Figure 2A). The capacity for differentiation into different mesenchymal tissues is one of the key properties of MSCs. The differentiation potential of hPDLSCs was evaluated by culturing them in osteogenic and adipogenic media. Small, round Alizarin Red-positive nodules and Oil Red-O-positive lipid droplets formed in the PDLSC cultures after three weeks of induction, indicating calcium accumulation and adipogenesis *in vitro* (Figure 2B). The presence of a cell surface marker is an important criterion for identifying stem cells. Therefore, we analyzed the surface markers of hPDLSCs, including CD29, CD90, CD34, and CD45 using flow cytometry. hPDLSCs exhibited positive expression of the mesenchymal stem cell surface markers CD90 (99.74%) and CD29 (92.23%); negative expression of the hematopoietic stem cell surface markers CD34 (0.06%) and CD45 (0.12%) (Figure 2C). The effect of metformin on cell proliferation was examined by performing a CCK-8 assay. The cell density increased from days 1 to 7, and no significant differences were observed among the 0, 30, 50, and 100 μM metformin-osteogenic groups from days 1 to 7 (*P*>0.05) (Figure 2D). Based on these results, metformin was not toxic to hPDLSCs, but it didn’t stimulate cell proliferation as well.

### Metformin promoted the osteogenic differentiation of hPDLSCs

**Figure 3:**
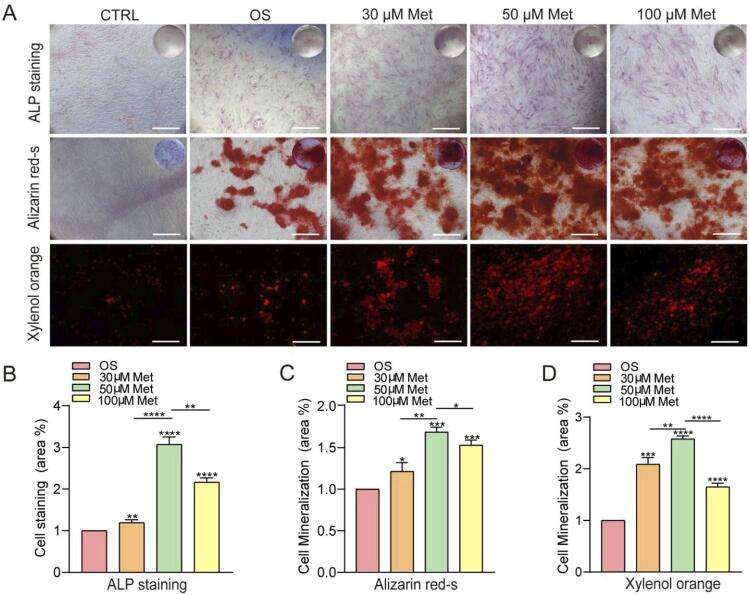
Metformin promotes the osteogenic differentiation of hPDLSCs. A, ALP staining, Alizarin red S staining, and xylenol orange staining showed that ALP activity and bone mineral nodule formation were increased in the metformin-treated group. (scale bar=500 μm). B, Area (%) of ALP staining. C, Area (%) of alizarin red S staining. D, Area (%) of xylenol orange staining. The values are presented as the means±SDs (n=3); *P<0.05, **P<0.01, ***P<0.001, ****P<0.0001

Cells were cultivated in osteogenic media with different concentrations of metformin to elucidate the effects of metformin on the osteogenic differentiation of hPDLSCs, and then we conducted staining with ALP, alizarin red S, and xylenol orange (Figure 3A). The area of stained cells (%) in the 50 μM metformin-osteogenic group was 3.1-fold higher than that of the osteogenic group (Figure 3B). The mineralized areas (%) of alizarin red S staining and xylenol orange staining in the 50 μM metformin-osteogenic group were 1.7-fold and 2.6-fold higher than those of the osteogenic induction group, respectively (*P*<0.01) (Figure 3C and D).

**Figure 4:**
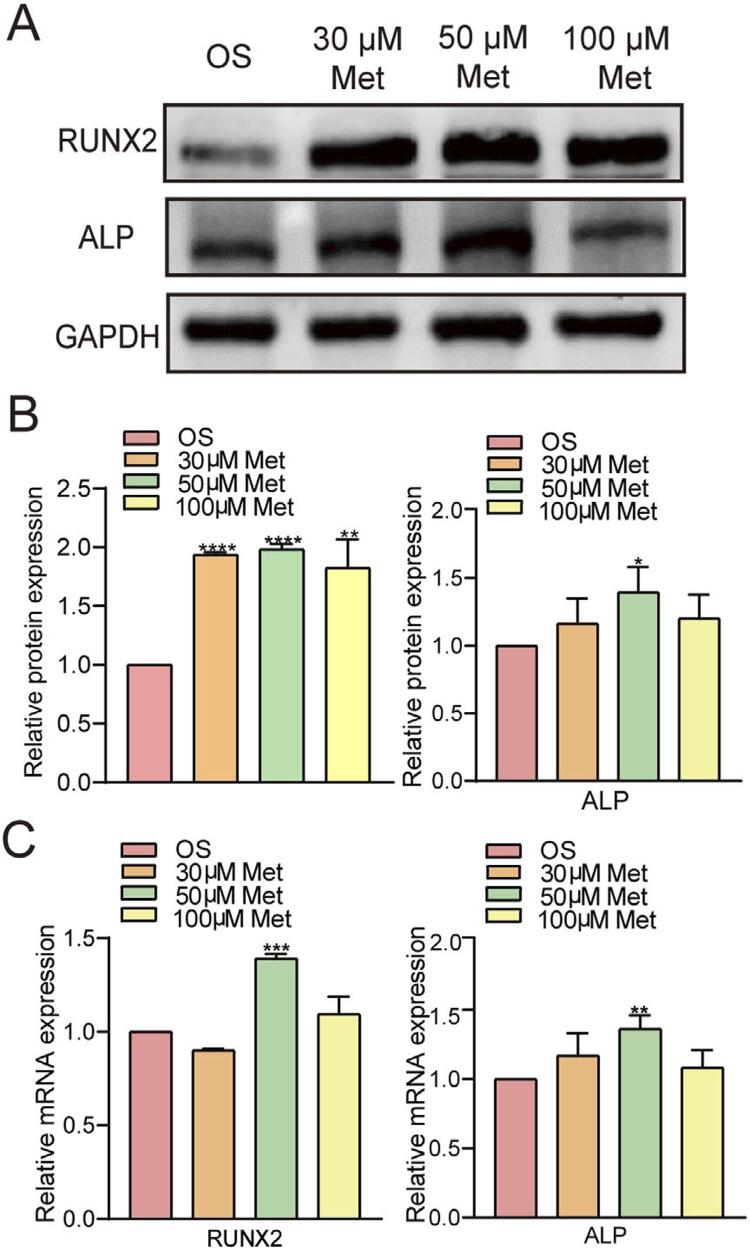
Metformin promotes osteogenic-related gene and protein expression in hPDLSCs. A and B, Western blot and quantitative analysis showing that the levels of mineralization-related proteins (RUNX2 and ALP) were increased by metformin. C, qRT-PCR showed that the levels of mineralization-related genes (RUNX2 and ALP) were increased by 50 μM metformin. The values are presented as the means±SDs. (n=3) *P<0.05, **P<0.01, ***P<0.001, ****P<0.0001

Western blot analyses showed higher levels of the RUNX2 (2.0-fold) and ALP (1.4-fold) proteins in the 50 μM metformin-osteogenic group than in the osteogenic induction group (*P*<0.05) (Figure 4A and B). The qRT‒PCR results showed 1.4-fold higher expression of the osteogenesis-associated markers *RUNX2* and *ALP* in the 50 μM metformin-osteogenic group than in the osteogenic induction group (*P*<0.01) (Figure 4C).

### Alginate-fibrin fibers encapsulating metformin enhanced the osteogenic differentiation of hPDLSCs

**Figure 5:**
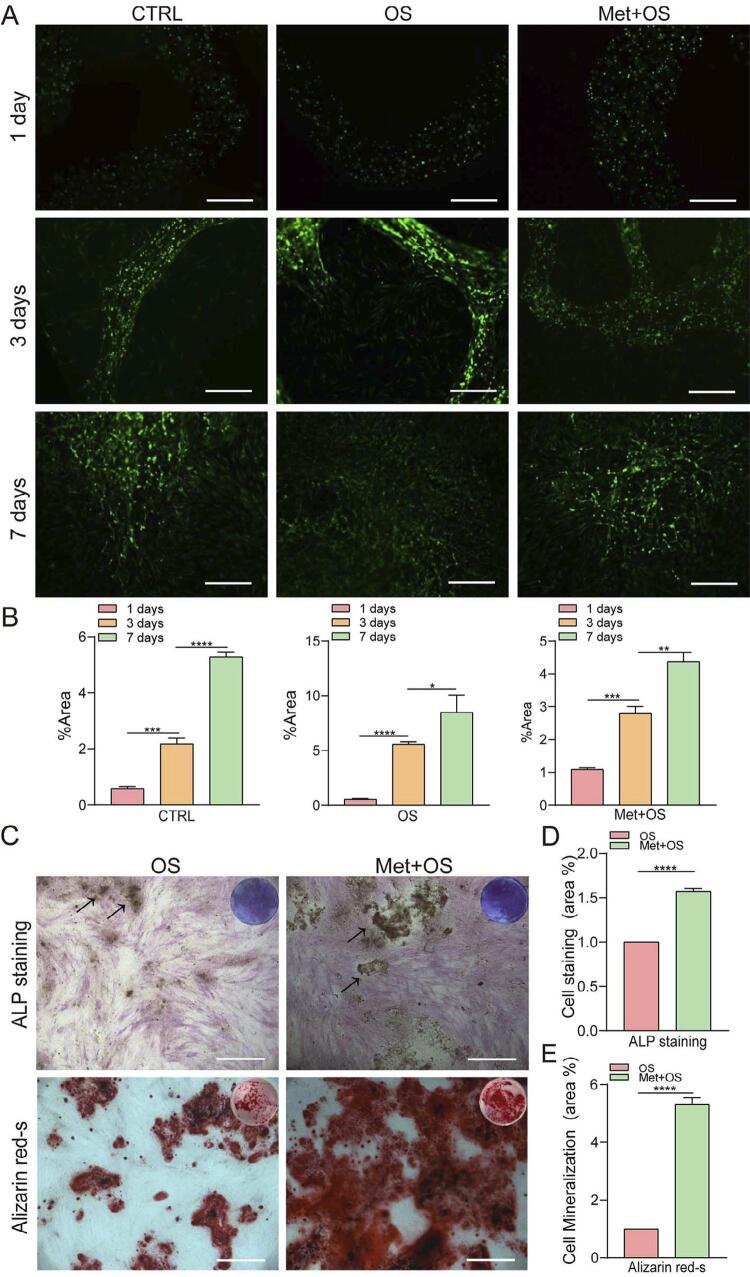
Alginate-fibrin fibers encapsulating hPDLSCs and Metformin. A, Green fluorescence indicated live cells, which number progressively increased from. The figure presents the days 1, 3, and 7, along with the gradual degradation of fibers. (Met+OS: alginate-fibrin fibers encapsulating hPDLSCs and 50 μM metformin, scale bar=500 μm) B, Area (%) of fluorescence. C, ALP staining and alizarin red S staining showed higher ALP activity and more bone mineral nodule formation in fibers encapsulating hPDLSCs with 50 μM metformin, compared to the cells alone (the black arrowhead shows the incomplete degraded part of the alginate-fibrin fibers, scale bar=500 μm). D, Area (%) of ALP staining. E, Area (%) of alizarin red S staining. The values are presented as the means±SDs. (n=3) *P<0.05, **P<0.01, ***P<0.001, ****P<0.0001

Live/dead cell staining revealed that hPDLSCs progressively migrated out from the alginate-fibrin fibers, the cell density significantly increased, with good cell development and proliferation, and the fiber structure gradually degraded from 3 to 7 days (Figure 5A and 5B). The results of ALP staining and alizarin red S staining showed that alginate-fibrin fibers encapsulating hPDLSCs and metformin (Met + OS group) further enhanced the osteogenic differentiation of hPDLSCs compared with the material carrying cells alone (OS group) (Figure 5C). The area (%) of ALP staining and the mineralization area (%) of alizarin red S staining in the Met + OS group were 1.6-fold and 5.3-fold higher than those in the OS group, respectively (*P*<0.0001) (Figure 5D and E). Overall, these results indicated that alginate-fibrin fibers loaded with metformin and hPDLSCs were promising materials for repairing periodontal bone defects.

### Activation of Shh/Gli1 in hPDLSCs by the metformin treatment

**Figure 6:**
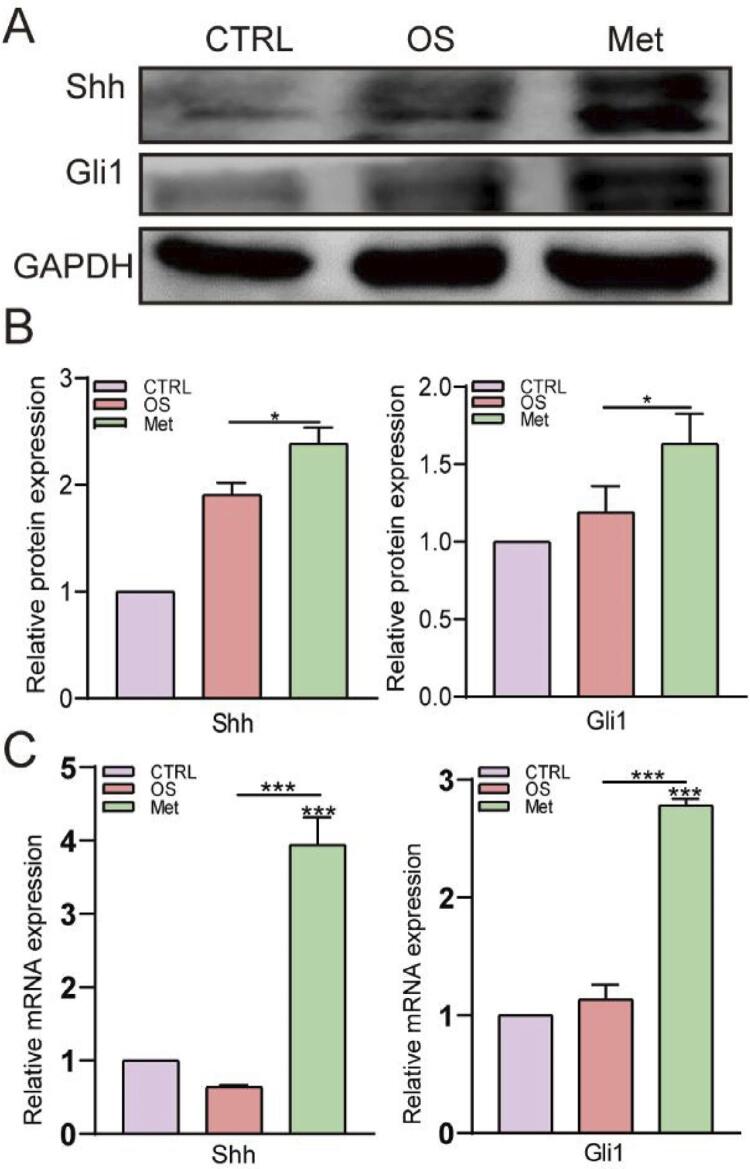
Metformin activated the Shh/Gli1 signaling pathway in hPDLSCs. A and B, Western blots and quantitative analyses showed that 50 μM metformin upregulated the expression of Shh/Gli1 proteins. C, qRT-PCR showed that metformin upregulated the expression of Shh/Gli1 genes. The values are presented as the means ± SDs. (n=3) *P<0.05, **P<0.01, ***P<0.001, ****P<0.0001

Shh/Gli1 is a classical signaling pathway that regulates tooth development and plays an active role in promoting the osteogenic differentiation of hPDLSCs. The transcription factor Gli1 is a well-known biomarker for Shh pathway activation. Shh and Gli1 protein levels in the 50 μM metformin-osteogenic group were upregulated 1.3-fold and 1.4-fold, respectively, compared to those in the osteogenic induction group, according to western blot assays (*P*<0.05) (Figure 6A and B). Furthermore, qRT‒PCR results revealed 6.0-fold and 3.0-fold increases in the expression of Shh and Gli1 in the 50 μM metformin-osteogenic group compared with the osteogenic group, respectively, indicating that metformin activated the Shh/Gli1 signaling pathway in hPDLSCs (*P*<0.001) (Figure 6C).

### Metformin promoted the osteogenic differentiation of hPDLSCs via the Shh/Gli1 signaling pathway

**Figure 7:**
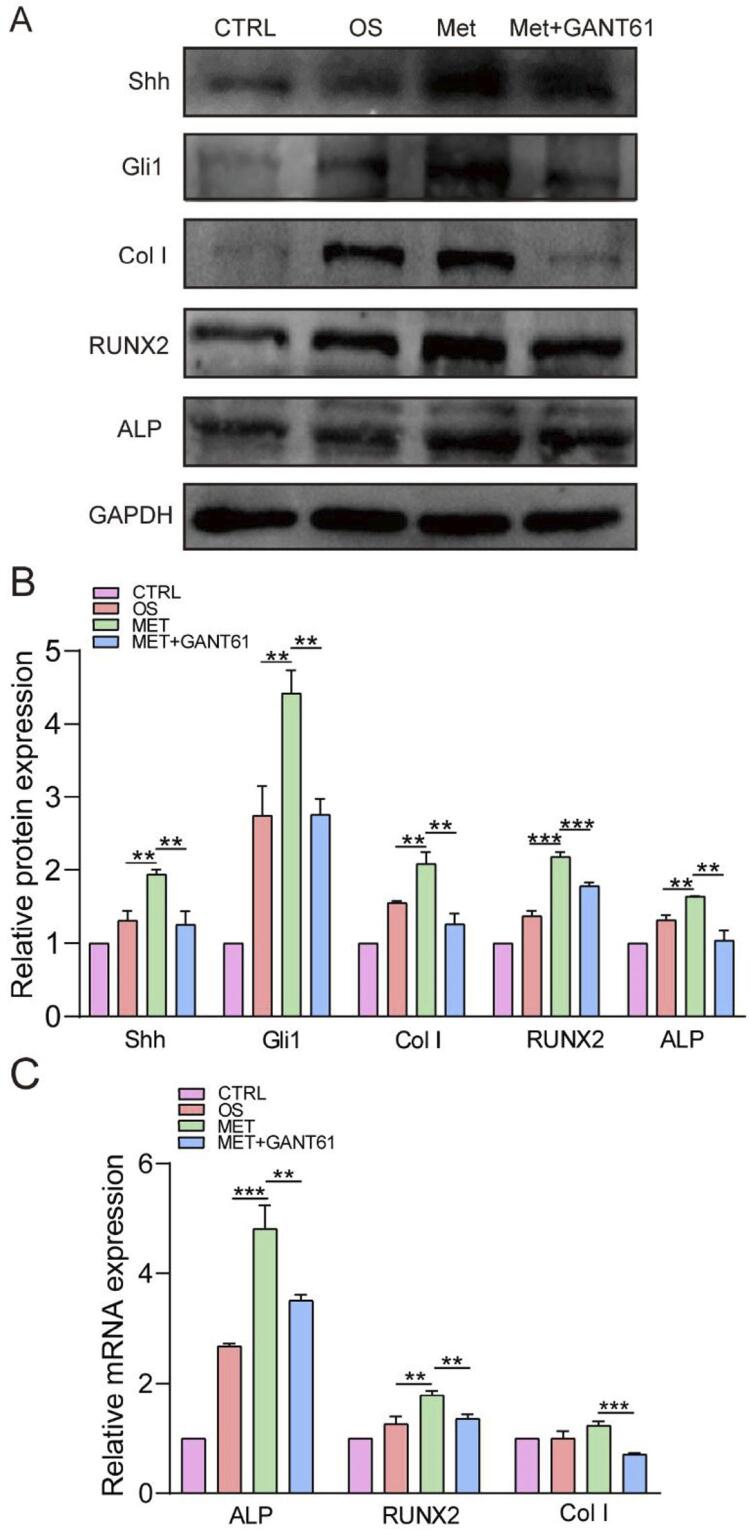
Shh/Gli1 activation was required for metformin to promote the osteogenic differentiation of hPDLSCs. A and B, Western blots and quantitative analyses showed that GANT61 inhibited the expression of Shh/Gli1 proteins, and the osteogenic differentiation related proteins (Col I, RUNX2 and ALP) were downregulated at the same time. C, qRT-PCR showed that GANT61 reduced the expression of osteogenic differentiation related genes (ALP, RUNX2 and Col I) that were upregulated by metformin. The values are presented as the means±SDs. (n=3) *P<0.05, **P<0.01, ***P<0.001, ****P<0.0001

Metformin activated the Shh/Gli1 signaling pathway in hPDLSCs under osteogenic induction conditions. To further investigate whether metformin regulated osteogenic differentiation via Shh/Gli1 pathway in hPDLSCs, we employed GANT61 as a selective inhibitor of Gli1/2. Firstly, GANT61 down-regulated the Shh and Gli1 protein expression by 1.6-fold compared to 50 μM metformin-osteogenic group, and decreased the expression of Col, RUNX2, and ALP by 1.7-fold, 1.2-fold, and 1.6-fold, respectively, in hPDLSCs, according to western blot (*P*<0.01) (Figure 7A and B). qRT-PCR provided similar results, in which the gene expressions of *ALP, RUNX2,* and *Col I* in metformin-GANT61 osteogenic group were downregulated 1.3 to 1.8 folds compared to metformin-osteogenic group, suggesting that the ability of osteogenesis induction by metformin in hPDLSCs could be reversed by GANT61 (*P*<0.01) (Figure 7C).

**Figure 8:**
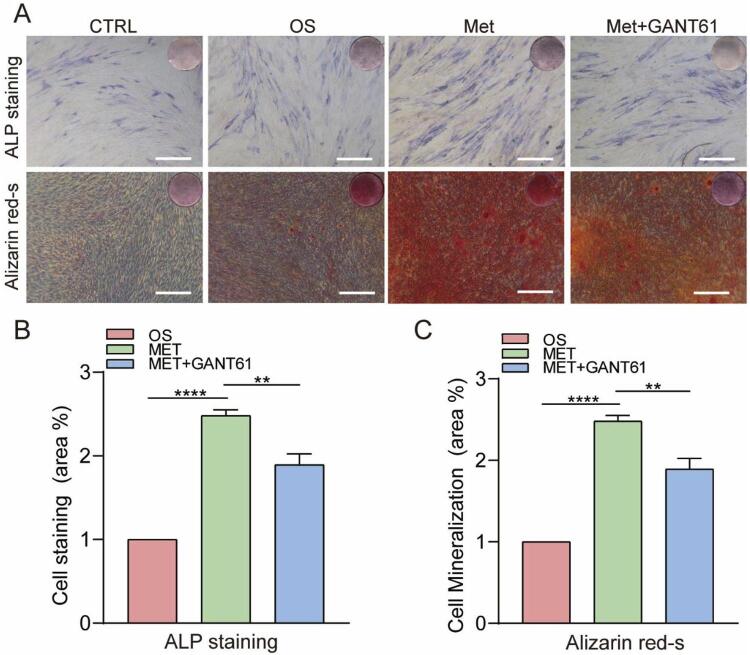
Inhibition of Shh/Gli1 decreased the osteogenic differentiation ability of hPDLSCs. A, ALP staining and alizarin red S staining showed that ALP activity and bone mineral nodule formation were decreased in the 50 μM metformin + GANT61 group (scale bar=500 μm). B, Area (%) of ALP staining. C, Area (%) of alizarin red S staining. The values are presented as the means±SDs. (n=3) *P<0.05, **P<0.01, ***P<0.001, ****P<0.0001

ALP staining and alizarin red S staining revealed decreases in ALP activity and the number of bone mineral nodules formed after treatment with GANT61 (Figure 8A). The area (%) of ALP staining and the mineralization area (%) of alizarin red S staining in the metformin-GANT61 osteogenic group were decreased by 1.3-fold and 1.6-fold compared to the metformin-osteogenic group, respectively (*P*<0.01) (Figure 8B and C). Collectively, these results suggest a role for metformin in promoting the osteogenic differentiation of hPDLSCs via the Shh/Gli1 pathway.

## Discussion

In this study, degradable alginate-fibrin hydrogel fibers that encapsulates metformin and hPDLSCs was produced for the first time. hPDLSCs showed good proliferation and osteogenic differentiation induced by metformin encapsulated in hydrogel fibers. In addition, metformin promoted a 3- to 6-fold upregulation of the Shh/Gli1 signaling pathway in hPDLSCs compared with the osteogenic group. Therefore, the novel alginate-fibrin-metformin-hPDLSCs fiber system displayed great potential in the repair and regeneration of periodontal bone tissues.

Metformin was nontoxic to the cells, but it didn’t stimulate cell proliferation as effectively. Zhao, et al.^18^ (2019) showed that metformin and hPDLSCs seeded on calcium phosphate cement scaffolds presented no effect on the proliferation of hPDLSCs. However, metformin combined with photobiomodulation therapy exerted a synergistic effect on promoting cell proliferation and reducing the production of reactive oxygen species in hPDLSCs pretreated with high glucose to simulate diabetes.^25^ Therefore, the authors speculated that metformin presents no effect on the proliferation of hPDLSCs under normal conditions and played a positive role in protecting cells from the damage of reactive oxygen species and promoting the proliferation of hPDLSCs under diabetic conditions. We used metformin at concentrations of 30 μM, 50 μM, and 100 μM to determine the suitable dosage of metformin that would enhance the osteogenic differentiation of hPDLSCs. ALP and alizarin red S staining revealed that 50 μM metformin induced the most significant increases in ALP activity and mineral deposition. qRT‒PCR and western blotting showed that 50 μM metformin was the most effective concentration at upregulating the expression of ALP and RUNX2; conversely, 100 μM metformin presented a lower expression of these proteins compared to 50 μM metformin. According to our findings, metformin considerably accelerated the osteogenic differentiation of hPDLSCs, with a more prominent effect found in a concentration of 50 μM metformin. Therefore, this concentration was used in all subsequent experiments. Zhang, et al.^26^ (2020) used metformin concentrations of 10 μM, 50 μM, and 100 μM and found that 50 μM metformin significantly promoted hPDLSCs migration and increased ALP activity and mineral deposition, consistent with our findings. In conclusion, the effects of metformin on hPDLSCs are limited to promoting cell osteogenesis by affecting cell differentiation rather than cell proliferation.

Metformin had difficulty reaching the local bone defect site after oral administration to achieve the purpose of regenerating alveolar bone defects. We constructed a novel calcium phosphate cement with metformin-loaded chitosan to promote the controlled release of metformin in our previous study.^27^ Shi, et al.^28^ (2018) developed an improved biphasic calcium phosphate combined with hPDLSCs for periodontal regeneration. These studies did not combine metformin and hPDLSCs. Therefore, in the present study, alginate-fibrin fibers with good biocompatibility and biosafety were prepared to deliver cells and release drugs in the bone defect area at the same time. Alginate-fibrin fibers present several advantages compared with systematic drug delivery. First, the local release of alginate-fibrin fibers carrying metformin avoids the adverse effects of drugs on the whole body. Second, the fibers release metformin and allow it to diffuse into the local microenvironment, producing a continuous induction effect on hPDLSCs.

Drug release and cell proliferation benefit from the good degradation properties of the material. Typically, the degradation of alginate hydrogels takes weeks or months. The addition of a small amount of fibrin significantly increases alginate hydrogel degradation. In our previous study, the addition of fibrin to oxidized alginate microbeads significantly increased the degradation of hydrogel and released the encapsulated cells from day 4.^22,29^ However, the volume of microbeads is too small to carry more cells and they are not conducive to fixation in the bone defect area. Therefore, we constructed oxidized alginate-fibrin in the form of fibers. Rapidly disintegrating fibers were generated in this study by incorporating a small amount of fibrin into the oxidized alginate and by adding the cell suspension with metformin. Our results showed that the fibers began to degrade on the third day and the released metformin promoted the osteogenic differentiation of hPDLSCs. Moreover, compared with microbeads, fibers formed larger pore canals after degradation, which facilitated the cellular uptake of oxygen and nutrients.^30^

In this experiment, prepared alginate-fibrin fibers encapsulated hPDLSCs and metformin at the same time. hPDLSCs showed good proliferation and differentiation abilities and metformin effectively induced osteogenesis. Alginate-fibrin fibers have a high potential for drug delivery and simulation of tissue morphology due to their fiber-like structure.^31^ Furthermore, alginate-fibrin fibers have good biocompatibility, biodegradability, hydrophilicity, injectability, and nontoxic properties^32,33^ and are expected to treat bone defect-related diseases. Maxillofacial bone defects are caused by trauma, craniofacial deformities, and tumors, resulting in dramatically decreased quality of life in affected individuals. Maxillofacial osseous defects are usually repaired by bone transplantation with either autologous or nonautologous substitutes.^34^ In recent years, an increasing number of studies have examined the combination of stem cells, drugs, and materials to treat bone defects. Alginate-fibrin fibers encapsulating hPDLSCs and metformin were expected to promote bone repair and regeneration by implanting them into the bone defect site using a syringe to achieve the delivery of hPDLSCs and the therapeutic effect of metformin. This study might lead to the development of a novel approach for treating alveolar bone loss caused by periodontitis. Alginate-fibrin fibers encapsulating hPDLSCs and metformin might be injected into the severe alveolar bone defect site in the process of periodontal surgery. hPDLSCs, as PDL-derived mesenchymal stem cells, can regenerate periodontal tissue. In addition, this biomaterial might be injected into the deep periodontal pocket to protect against bone loss in individuals with early periodontitis. The healing process of the bone and periodontium in diabetic patients depends on the level of glycemic control.^35^ Thus, alginate-fibrin fibers encapsulating hPDLSCs and metformin also have potential applications in diabetic bone and periodontium regeneration.

Many studies have investigated the mechanism by which metformin promotes osteogenic cell differentiation. Metformin was reported to stimulate the osteogenic differentiation of MC3T3E1 cells via the transactivation of RUNX2 by the AMPK/USF-1/SHP regulatory cascade, and the activation/redistribution of ERK-1/2 and induction of e/iNOS activity might also participate in this mechanism.^36,37^However, the related mechanism of the osteogenic differentiation of hPDLSCs by metformin was unclear. Metformin promoted the osteogenesis of hPDLSCs by upregulating the Akt/Nrf2 signaling pathway and protecting cells from oxidative stress, according to Jia, et al.^8^ (2020). The osteogenic effect of metformin was inhibited by administering LY294002, an inhibitor of Akt phosphorylation.^8^ According to our findings, metformin upregulated the expression of Shh/Gli1 by 3- to 6-fold in hPDLSCs. Metformin promoted pulmonary vascular development in hyperoxic newborn mice by upregulating the expression of Gli1 in pulmonary vascular endothelial cells.^38^ In addition, the protective effect of metformin on the endothelium under hyperglycemic conditions might be ascribed in part to its activation of Shh, which inhibits autophagy.^39^ However, researchers have not reported whether metformin promotes osteogenic differentiation via the Shh/Gli1 signaling pathway.

We further explored the relationship between the increased expression of Shh/Gli1 and osteogenesis in hPDLSCs. The capacity of metformin to promote the osteogenic differentiation of hPDLSCs was decreased by 1.2- to 1.7-fold compared to the osteogenic induction group when the Shh/Gli1 signaling pathway was downregulated by 1.6-fold, as evidenced by the administration of GANT61, a selective transcriptional inhibitor of Gli1. Based on these findings, we suggest that Shh/Gli1 signaling is involved in the metformin-mediated enhancement of osteogenesis in hPDLSCs. Previous studies by our group also revealed that the Shh signaling pathway played an important role in regulating the osteogenic differentiation of DPSCs.

In addition, many studies have shown that the Shh signaling pathway plays an important role in regulating tooth growth and development and stem cell differentiation.^40^ Shh derived from the dental epithelium regulates dental mesenchymal stem cells during embryonic development. Gli1^+^ cells in rat incisors and peripheral neurovascular bundles were identified as mesenchymal stem cells.^41^ Shh was expressed at significantly higher levels in the middle of the alveolar fossa 3 days after tooth extraction, suggesting that it might act on mesenchymal stem cells and bone-producing cells to promote trabecular development in the early stage of alveolar fossa healing.^42^ Therefore, we hypothesized that metformin could enhance the aggregation and differentiation of mesenchymal stem cells by upregulating the Shh/Gli1 pathway, which requires further exploration in the future.

## Conclusions

This study developed novel constructs consisting of degradable alginate-fibrin fibers encapsulating and delivering hPDLSCs and metformin, and determined the regulatory role of the Shh/Gli1 signaling pathway in the metformin-induced osteogenic differentiation of hPDLSCs for the first time. The constructs, including metformin, were biocompatible and were not toxic to hPDLSCs. Metformin substantially enhanced the osteogenesis of hPDLSCs, with highly elevated ALP, RUNX2, and Col I expression. Degradable alginate-fibrin hydrogel fibers encapsulating metformin and hPDLSCs showed excellent cell activity and osteogenic differentiation. The Shh/Gli1 signaling pathway was upregulated and affected the metformin-induced osteogenesis of hPDLSCs. When the Shh/Gli1 signaling pathway was inhibited in metformin-treated hPDLSCs, ALP activity, bone mineral nodule formation, and osteogenic markers were decreased. The degradable alginate-fibrin fibers encapsulating and delivering hPDLSCs and metformin are promising for dental, periodontal, and bone regeneration applications.
